# Supplementary material for: Iterative improvement in the automatic modular design of robot swarms
Source: PeerJ Comput Sci. 2020 Dec 7;6:e322. doi: 10.7717/peerj-cs.322 (PMC7924708; doi:10.7717/peerj-cs.322)
Supplement: Supplemental Information 3 [file peerj-cs-06-322-s003.zip › argos3/doc/api/standalone/a00567_source.html]

ARGoS: plugins/simulator/entities/cylinder\_entity.h Source File


- Main Page
- Related Pages
- Namespaces
- Classes
- Files

- File List
- File Members

# plugins/simulator/entities/cylinder\_entity.h

Go to the documentation of this file.

```
00001 
00008 #ifndef CYLINDER_ENTITY_H
00009 #define CYLINDER_ENTITY_H
00010 
00011 namespace argos {
00012    class CCylinderEntity;
00013    class CEmbodiedEntity;
00014    class CLEDEquippedEntity;
00015 }
00016 
00017 #include <argos3/core/simulator/entity/embodied_entity.h>
00018 #include <argos3/core/simulator/entity/composable_entity.h>
00019 #include <argos3/plugins/simulator/entities/led_equipped_entity.h>
00020 
00021 namespace argos {
00022 
00023    class CCylinderEntity : public CComposableEntity {
00024 
00025    public:
00026 
00027       ENABLE_VTABLE();
00028 
00029       CCylinderEntity();
00030 
00031       CCylinderEntity(const std::string& str_id,
00032                       const CVector3& c_position,
00033                       const CQuaternion& c_orientation,
00034                       bool b_movable,
00035                       Real f_radius,
00036                       Real f_height,
00037                       Real f_mass = 1.0f);
00038 
00039       virtual void Init(TConfigurationNode& t_tree);
00040       virtual void Reset();
00041 
00042       /*
00043        * Enables the LEDs for this entity.
00044        * Adds the LED equipped entity to the given medium.
00045        * If you don't call this method, the LEDs added with
00046        * CCylinderEntity::AddLED() won't be update correctly.
00047        * @param c_medium The medium to which the LEDs must be associated.
00048        * @see CCylinderEntity::AddLED()
00049        */
00050       void EnableLEDs(CLEDMedium& c_medium);
00051 
00052       /*
00053        * Disables the LEDs for this entity.
00054        */
00055       void DisableLEDs();
00056 
00065       void AddLED(const CVector3& c_offset,
00066                   const CColor& c_color = CColor::BLACK);
00067 
00068       inline CEmbodiedEntity& GetEmbodiedEntity() {
00069          return *m_pcEmbodiedEntity;
00070       }
00071 
00072       inline CLEDEquippedEntity& GetLEDEquippedEntity() {
00073          return *m_pcLEDEquippedEntity;
00074       }
00075 
00076       inline Real GetRadius() const {
00077          return m_fRadius;
00078       }
00079 
00080       inline void SetRadius(Real c_radius) {
00081          m_fRadius = c_radius;
00082       }
00083 
00084       inline Real GetHeight() const {
00085          return m_fHeight;
00086       }
00087 
00088       inline void SetHeight(Real c_height) {
00089          m_fHeight = c_height;
00090       }
00091 
00092       inline Real GetMass() const {
00093          return m_fMass;
00094       }
00095 
00096       inline void SetMass(Real f_mass) {
00097          m_fMass = f_mass;
00098       }
00099 
00100       virtual std::string GetTypeDescription() const {
00101          return "cylinder";
00102       }
00103 
00104  private:
00105 
00106       CEmbodiedEntity*    m_pcEmbodiedEntity;
00107       CLEDEquippedEntity* m_pcLEDEquippedEntity;
00108       Real                m_fRadius;
00109       Real                m_fHeight;
00110       Real                m_fMass;
00111       CLEDMedium*         m_pcLEDMedium;
00112 
00113    };
00114 
00115 }
00116 
00117 #endif
```

---

Generated on 10 Jul 2018 for ARGoS by 
 1.6.1
